# How Do Amoebae Swim and Crawl?

**DOI:** 10.1371/journal.pone.0074382

**Published:** 2013-09-11

**Authors:** Jonathan D. Howe, Nicholas P. Barry, Mark S. Bretscher

**Affiliations:** Cell Biology Division, Medical Research Council Laboratory of Molecular Biology, Cambridge, Cambridgeshire, United Kingdom; University of Birmingham, United Kingdom

## Abstract

The surface behaviour of swimming amoebae was followed in cells bearing a cAR1-paGFP (cyclic AMP receptor fused to a photoactivatable-GFP) construct. Sensitized amoebae were placed in a buoyant medium where they could swim toward a chemoattractant cAMP source. paGFP, activated at the cell’s front, remained fairly stationary in the cell’s frame as the cell advanced; the label was not swept rearwards. Similar experiments with chemotaxing cells attached to a substratum gave the same result. Furthermore, if the region around a lateral projection near a crawling cell’s front is marked, the projection and the labelled cAR1 behave differently. The label spreads by diffusion but otherwise remains stationary in the cell’s frame; the lateral projection moves rearwards on the cell (remaining stationary with respect to the substrate), so that it ends up outside the labelled region. Furthermore, as cAR1-GFP cells move, they occasionally do so in a remarkably straight line; this suggests they do not need to snake to move on a substratum. Previously, we suggested that the surface membrane of a moving amoeba flows from front to rear as part of a polarised membrane trafficking cycle. This could explain how swimming amoebae are able to exert a force against the medium. Our present results indicate that, in amoebae, the suggested surface flow does not exist: this implies that they swim by shape changes.

## Introduction

The mechanism by which animal cells crawl is moot, although there is a consensus that the same basic mechanism is used by most eukaryotic cells. We recently discovered that *Dictyostelium discoideum* amoebae, as well as human polymorphonuclear leukocytes (PMNs), can swim towards a chemotactic source in a buoyant medium containing Ficoll [1]. The more detailed studies on amoebae showed that small lateral projections on the sides of these cells remained stationary with respect to the medium, moving down the cell as it moved forwards. This implied that these projections are not acting as paddles to push the cell forward. The central question which those studies raised was: how do these cells exert force against the medium in order to advance? We tentatively concluded that the cell’s surface must be flowing backwards, from the leading front towards the cell’s rear, and that this flow could provide the traction against the medium required to advance the cell. This suggestion did not fit with earlier results which indicated that such a flow does not exist in amoebae migrating on a solid substratum [2].

Here we try to gain a better understanding of how these cells move and, in particular, search for a surface flow in swimming and in crawling amoebae. Like Traynor et al. [2], we cannot find any evidence for such a flow in amoebae and conclude that, unlike in human giant HeLa cells [3] or avian fibroblasts [4] a polarised endocytic cycle appears not to exist.

## Materials and Methods

### Cells

Ax2 expressing cAR1-GFP or cAR1-paGFP [2] were provided by David Traynor and grown and studied at 22°C. The cells were sensitized to cAMP by washing with KK2 buffer (16.5 mM KH_2_PO_4_, 3.8 mM K_2_HPO_4_, 2 mM MgSO_4,_ 0.1 mM CaCl_2_), starving them for 1 hour and then pulsing them with cAMP [2] for 3.75 or 3.0 hours respectively and finally washing them in KK2 buffer without CaCl_2_.

### Chemotaxis

The chamber and basic arrangement used to study swimming has been described previously [1]. In brief, the chamber contained 30 µl of ~15% Ficoll 400 containing 2x10^4^ amoebae; these migrated towards a chemoattractant source in the tip of a needle. Details of how needles were prepared are included in Supporting Information (see Protocol S1). Cells chemotaxing on a substrate were observed on a No. 2 coverslip with a Dunn chamber.

### Microscopy

**Figure 1:**
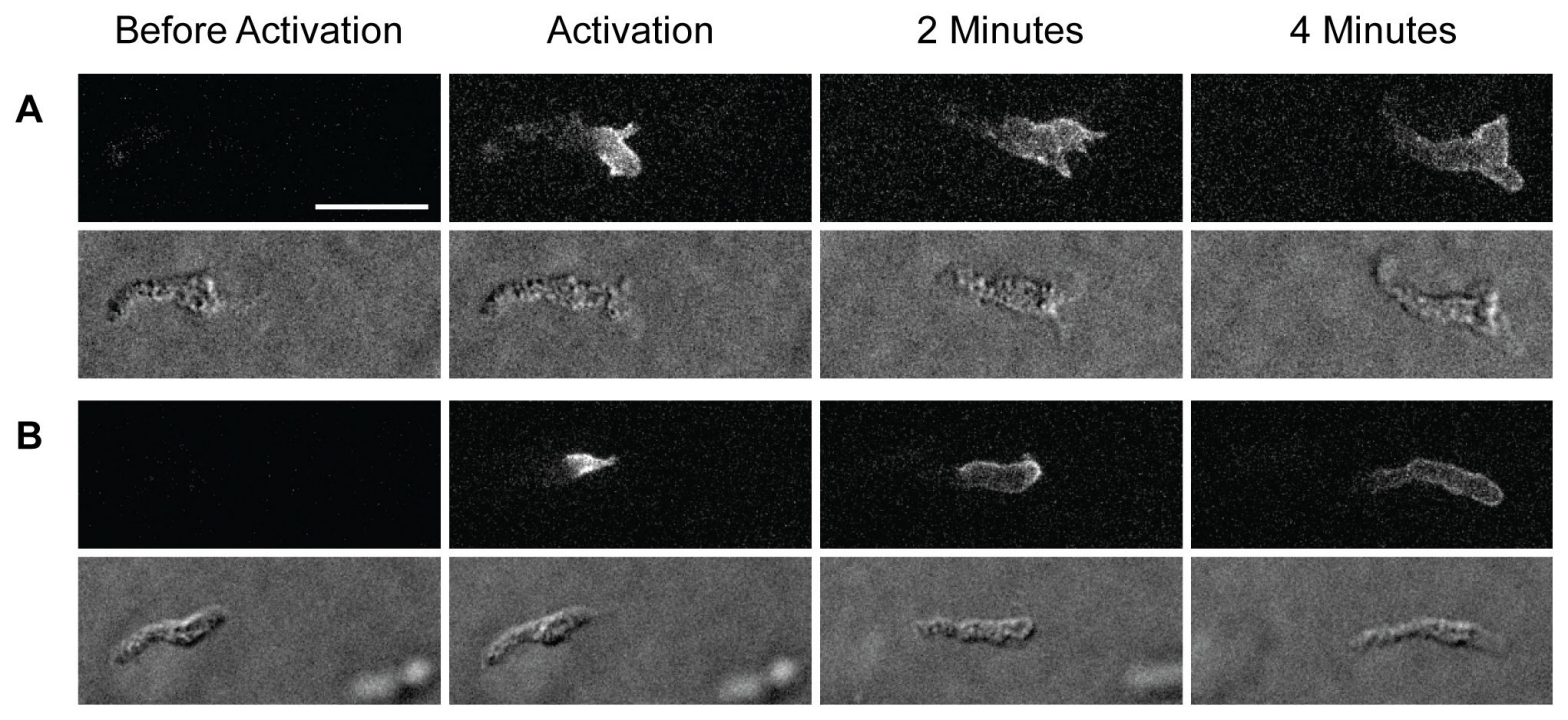
Lack of rearward surface flow in swimming cells. Two cAR1-paGFP expressing cells (A, B) in which the front has been photoactivated swimming toward a cAMP source (on the right in all figures). Above, fluorescence images taken with 488 nm laser excitation and GFP filters; below, corresponding DIC images. From left to right: before photoactivation by a brief pulse at 405 nm, immediately after activation and 2 or 4 minutes later. Scale bar here and elsewhere, 20 µm.

Cells were observed using an Andor Revolution XD spinning disk confocal system built around a Nikon Eclipse Ti microscope with a 60x water objective. Z-stacks of 11 images 1 µm apart were taken each 5 seconds (except for Figure 1 which had a 2 minute time interval) with a 488 nm laser and gfp filters. Photoactivation of paGFP was achieved by point scanning with a 405 nm laser using a 50-200 µsec pixel dwell time. Images were captured with an Andor iXon EMCCD camera and processed using Fiji software. Images of cAR1-paGFP expressing cells before activation are presented in the figures by a single image through the cell’s mid-plane; other images are z-projections of between 4 and 8 z-planes that encompass the full depth of the cell throughout the acquisition. Consequently the background in the fluorescence images before activation is apparently lower than after activation.

## Results

The absence of any rearward membrane flow in substrate attached amoebae was originally deduced by labelling a spot on the surface of an amoeba and observing whether this spot is swept backwards [2] as the cell moves forward. The μm-sized spot, located on the dorsal surface near the cell’s front, was induced either by photobleaching a cAR1-GFP labelled amoeba or by photoactivating a cAR1-paGFP cell. In either case the window for observation was limited to about 20 seconds before the coherence of the spot was lost by diffusion. In both cases, the observed spot remained in about the same place with respect to the front of the cell: no evidence for a rearward flow of the cell’s surface could be detected. We have examined this same question using cAR1-paGFP cells which are swimming towards a source of chemoattractant. To provide a larger time window, we photoactivated the front of a cell and watched this over a period of a few minutes. The results for two cells are presented in Figure 1; this shows that the label remains at the front of the cell for an extended period with no indication that unlabelled membrane is added at the front as the cell advances.

**Figure 2:**
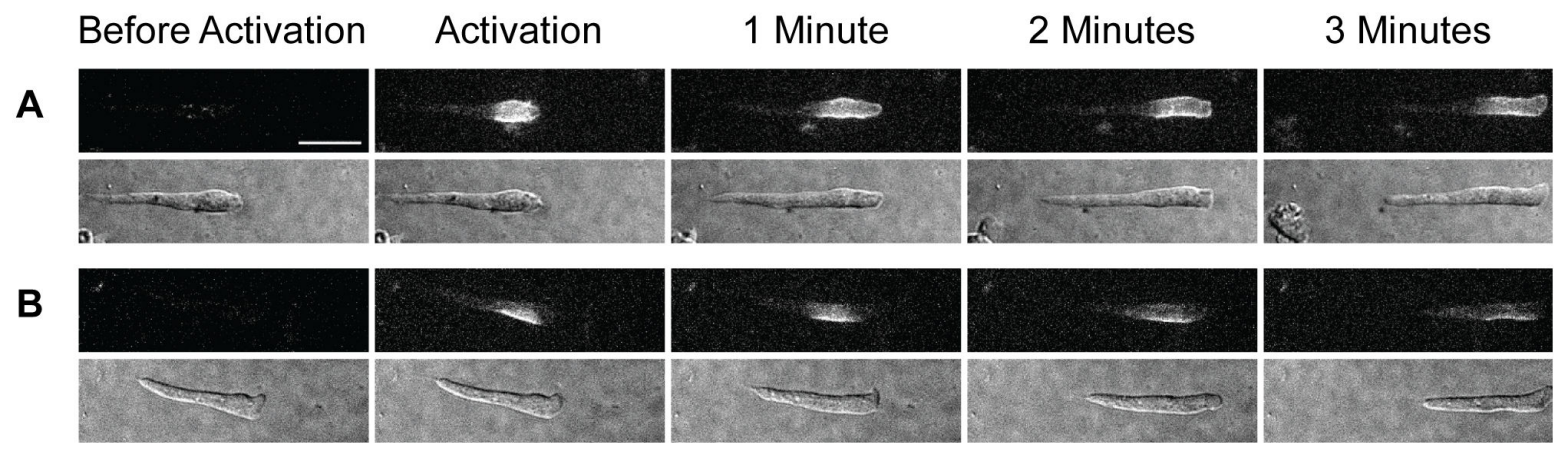
Lack of rearward surface flow in crawling cells. Two cAR1-paGFP expressing cells in which the front (A) or lateral side near the front (B) has been photoactivated crawling toward a cAMP source. Fluorescence and DIC images taken as in Figure 1. From left to right: before photoactivation, immediately after activation and 1, 2 or 3 minutes later.

We have repeated this approach with cells chemotaxing on a substrate (Figure 2): a region just behind the cell’s leading edge (A) was activated as above, or a side region near the front (B). In both cases the labelled surface marker spread along the surface by diffusion but remained in the same region of the cell as the cell advanced.

**Figure 3:**
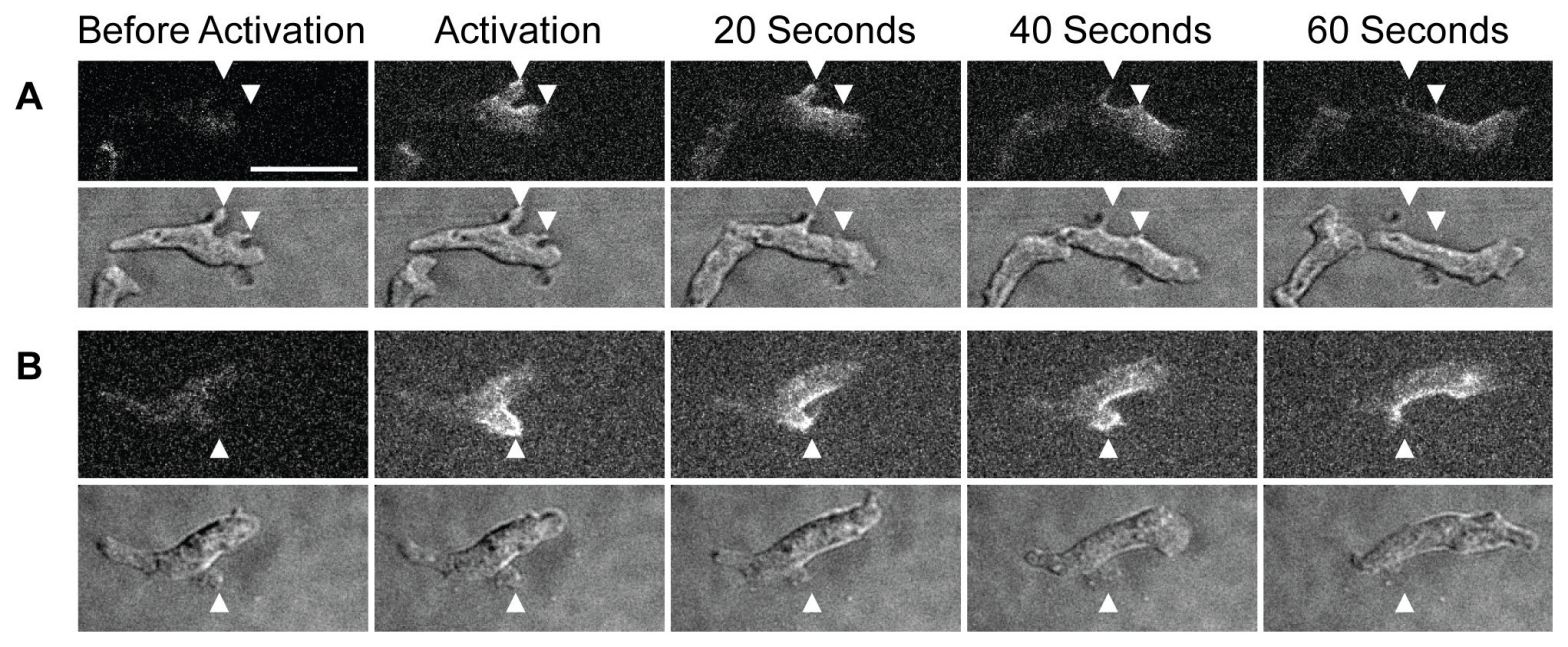
Lateral processes and surface markers move differently as cells crawl. Two cAR1-paGFP expressing cells (A, B) in which (A) two processes or (B) one process near the front of the cell have been photoactivated crawling toward a cAMP source. Fluorescence and DIC images taken as in Figure 1. From left to right: before photoactivation, immediately after activation and 20, 40 or 60 seconds later. Arrow tips indicate positions of the projections, which remain stationary with respect to the substratum.

Lateral projections on swimming or crawling amoebae are known to move backwards as the cell advances: this rearward movement might be effected by a rearward flow of membrane in the cell’s surface. We therefore tried to photoactivate a forming projection on the surface of a crawling cell and see how it behaved compared to the marked surface. Two examples are shown in Figure 3. They show that as the cell advances, the projection remains stationary with respect to the substratum, whereas the surface label advances with the cell such that the two become almost separated.

**Figure 4:**
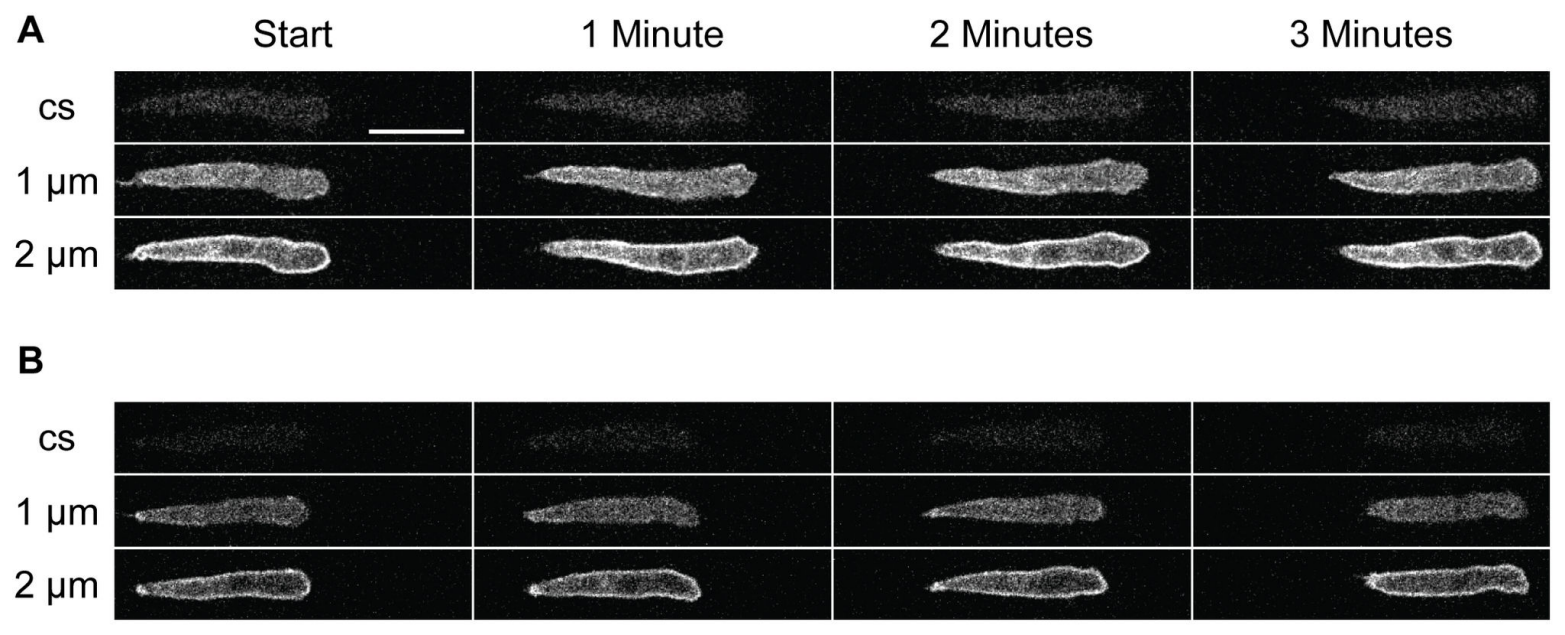
Crawling cells can advance with only minor snaking. Two cAR1-GFP expressing cells (A, B) crawling toward a cAMP source. Fluorescence images taken as in Figure 1 and presented at 1 minute intervals. Three images for each time point are shown: these capture the base of the cell on the coverslip (cs), and 1 µm or 2 µm above that. Images were taken each 5 seconds; the complete data set can be seen as Video S1 and Video S2 in Supporting Information. The cells move with little lateral motion except at the front of each cell.

When amoebae swim their bodies usually have an irregular shape which changes with time and which might effect this movement [5]: we refer to this movement as snaking. When these cells chemotax on a substratum, they frequently move for short distances, whilst maintaining a linear shape. We therefore looked for such cells which move a whole cell’s length to see whether they can do so. Figure 4 shows two cells whose surfaces are uniformly labelled with cAR1-GFP crawling on a substratum; a z-stack of fluorescence images at a separation of 1 µm was captured as the cells advance. Examination of the images closest to the substrate does not indicate any visible snaking in the z-axis (see Supporting Information; Video S1 and Video S2). This suggests that it is possible for a cell to move without substantial snaking motions of its main body length. It may also be noted that the cells shown in Figure 2 are also fairly linear.

Photoactivation of cAR1-paGFP with a 405nm laser beam might cause damage to the part of the cell being irradiated. When this strain or its parent, Ax2, is given a whole body irradiation with the same intensity used for photoactivation, the cells usually stop moving and may round up somewhat. However, after a few minutes of aberrant behaviour they start chemotaxing again (not shown). This radiation can clearly have a short-term deleterious effect on a cell’s movement; however, this is usually reversed and the cells continue to migrate as before. We do not think this effect has any bearing on the results presented above since we only activate a relatively small portion of the cell.

## Discussion

A chemotaxing amoeba is a particularly suitable test organism for detecting any flow in its surface membrane for two reasons: (a) amoebae move fairly swiftly with directional persistence and (b) because the diffusion of its surface proteins is unusually low (2.7x10^-10^ cm^2^/sec, as measured for cAR1 [6]). We sought to discover whether membrane from the rear regions of a migrating amoeba is recycled through the cell and added at the cell’s front as it advances and which, as a consequence, would cause a rearward surface flow away from the cell’s front. We could find no evidence for such membrane addition and conclude that no such flow exists in swimming or crawling cells, in line with earlier measurements on crawling cells [2]. This lack of flow is clearly demonstrated for two cells in which a surface projection near the cell’s front can be followed (Figure 3): the projection, initially in the centre of induced fluorescence, moves rearward on the cell, leaving the centre of fluorescence in the same place in the cell’s frame.

These results raise many difficult questions, not the least being how these amoebae swim. Our previous observations on swimming amoebae [1] led us to conclude that lateral projections could not act as paddles to provide a thrust against the medium. We tentatively concluded that a rearward surface membrane flow might provide the propulsive force against the medium which is needed for motion. However, this appears not to be the case and we are left to conclude that amoebae must pass through a series of shape changes which enable them to push somehow against the viscosity of the medium [5]. Swimming amoebae and those crawling on a substratum are often superficially similar in appearance and we tend to imagine that they swim and move on a surface using the same basic processes. If this is so, we are left to conclude that the only way they can swim, or move on a substrate, is by some form of shape changes. However, when these cells are (more easily) observed as they move on a substratum, they can do so for some distance without much deviation from a rod-like form (Figure 4): this suggests that they do not crawl, or therefore swim, by snaking. Simply put, we do not understand how these cells swim, and therefore how they move.

In several polarised eukaryotic cells the membrane endocytosed by coated pits is returned to the cell surface membrane at the front of the cell; this is best demonstrated for avian fibroblasts [4], COS cells [7], giant HeLa cells [3,8] and shmooing and budding yeast cells [9]. Given the rate at which an amoeba internalises its surface [2,10] a large rearward flow of surface membrane would be expected if this membrane were exocytosed at the cell’s front. The present experiments clearly suggest that this is not the case in migrating amoebae. However, our experiments do not preclude the existence of a localised endo/exocytic cycle.

We conclude that amoebae have no overt membrane flow and that, of the possible modes by which they might swim, we could find no evidence in favour of any of them.

## Supporting Information

Protocol S1 **Needle preparation.** Description of needle preparation for chemotactic swimming chamber.
(DOCX)

Video S1 **Crawling rod-like cell from Figure 4A.** Complete data set from which Figure 4A was prepared. Presented as a movie in which 3 minutes are compressed to 6 seconds.
(AVI)

Video S2 **Crawling rod-like cell from Figure 4B.** Complete data set from which Figure 4B was prepared. Presented as a movie in which 3 minutes are compressed to 6 seconds.
(AVI)
