# Supplementary material for: How Do Amoebae Swim and Crawl?
Source: PLoS One. 2013 Sep 11;8(9):e74382. doi: 10.1371/journal.pone.0074382 (PMC3770602; doi:10.1371/journal.pone.0074382)
Supplement: Protocol S1 — Needle preparation. Description of needle preparation for chemotactic swimming chamber. (DOCX) [file pone.0074382.s001.docx]

**Protocol S1. Needle preparation**.

Harvard borosilicate capillaries (GC120F-15) were pulled using a Sutter micropipette puller. Each needle was placed in a holder (made from a 3 cm piece of a pasteur pipette, broken so that one end was fluted and the other end, about 1 mm diameter, sealed), making sure not to touch the tip. These were laid horizontally with their open ends dipping into 50 μl of a solution of 0.5% agarose (Biogene, cat. no. 300-200) and 0.1% Tween in water, in a humidified chamber at about 50°C. The agarose solution creeps to the very tip of the sealed end of the needle and the process stopped when the thicker part of the needle had a column of ~0.5-2.5 mm of agarose. The needle was then moved to room temperature (where the agarose solidifies) and examined to remove any needles with air locks in the agarose (about 10-50%). The open end of each needle was then filled with a holding solution (1 mM EDTA, 0.1% Tween, 1 mM azide) and stored in a humidified box. Before use, the needle tip was broken to expose an opening of about 10 μm; this broken tip was immediately placed in a water-filled holder. In some cases the needle was tested by placing the needle in a holder containing a ~50% saturated solution of bromophenol blue (at about pH 7) and held in that for 1-2 days, the tip then removed from the holder, washed in water and quickly examined carefully to see whether any visible dye had entered. If it hadn't, the needle was rejected. To use, the tip was placed in a holder containing 0.1 mM cAMP in water and the open end filled with the same and held in the humidified box for 24h hours. Before its actual use, it was removed from the holder and the tip washed to remove any cAMP solution.
